# Initiating pollen sensitization – complex source, complex mechanisms

**DOI:** 10.1186/s13601-020-00341-y

**Published:** 2020-08-31

**Authors:** Lisa Pointner, Athanasios Bethanis, Michael Thaler, Claudia Traidl-Hoffmann, Stefanie Gilles, Fatima Ferreira, Lorenz Aglas

**Affiliations:** 1 Department of Biosciences, University of Salzburg, Hellbrunnerstraße. 34, 5020 Salzburg, Austria; 2 Chair and Institute of Environmental Medicine, UNIKA-T, Technical University of Munich and Helmholtz Zentrum München, Augsburg, Germany; 3 Christine-Kühne-Center for Allergy Research and Education (CK-Care), Davos, Switzerland

**Keywords:** Allergy, Pollen, Sensitization, Allergens, Th2 polarization, Alarmins, Adjuvants, IL-4, Innate immunity, Dendritic cells

## Abstract

The mechanisms involved in the induction of allergic sensitization by pollen are not fully understood. Within the last few decades, findings from epidemiological and experimental studies support the notion that allergic sensitization is not only dependent on the genetics of the host and environmental factors, but also on intrinsic features of the allergenic source itself. In this review, we summarize the current concepts and newest advances in research focusing on the initial mechanisms inducing pollen sensitization. Pollen allergens are embedded in a complex and heterogeneous matrix composed of a myriad of bioactive molecules that are co-delivered during the allergic sensitization. Surprisingly, several purified allergens were shown to lack inherent sensitizing potential. Thus, growing evidence supports an essential role of pollen-derived components co-delivered with the allergens in the initiation of allergic sensitization. The pollen matrix, which is composed by intrinsic molecules (*e.g.* proteins, metabolites, lipids, carbohydrates) and extrinsic compounds (*e.g.* viruses, particles from air pollutants, pollen-linked microbiome), provide a specific context for the allergen and has been proposed as a determinant of Th2 polarization. In addition, the involvement of various pattern recognition receptors (PRRs), secreted alarmins, innate immune cells, and the dependency of DCs in driving pollen-induced Th2 inflammatory processes suggest that allergic sensitization to pollen most likely results from particular combinations of pollen-specific signals rather than from a common determinant of allergenicity. The exact identification and characterization of such pollen-derived Th2-polarizing molecules should provide mechanistic insights into Th2 polarization and pave the way for novel preventive and therapeutic strategies against pollen allergies.

## Background

Worldwide, the sensitisation rate to pollen allergens is around 40% and over 400 million people suffer from allergic rhinitis symptoms caused by pollinosis [1–4]. Wind plays a key role in the induction of pollen sensitization enabling the direct contact of pollen with the human immune system at sites such as the upper respiratory tract, the ocular and oral mucosal surfaces, where the humid milieu facilitates the release of soluble allergens and other co-delivered bioactive compounds from the pollen matrix. Among wind-pollinated plants, the four plant families Oleaceae, Poaceae, Asteraceae and Betulaceae represent the main sources of allergenic pollen in Europe [5, 6]. Up to now, 987 different allergens have been officially described, of which 195 are registered as plant-derived airborne allergens (https://www.allergen.org, 1st April 2020). Besides triggering seasonal rhinoconjunctivitis symptoms, a clinical condition also known as “hay fever”, pollen can also cause asthma, skin inflammation, and even food allergies due to structural homology of food antigens to some pollen allergens [7–14]. The rising numbers of pollen-affected individuals, the variety of symptoms and the impact on the patients´ quality of life are making pollen allergies a vast and serious socio-economic burden of Western civilization [15]. In this respect, it is of utmost importance to understand the underlying mechanisms of sensitization to pollen allergens in order to develop innovative therapeutic strategies to efficiently tackle pollinosis. The scope of this review is to summarize the current concepts and newest advances in research focusing on pollen sensitization.

## Current concepts of allergic sensitization

Exposure to environmental proteins does not normally trigger an immune response due to their non-pathogenic nature, yet allergic sensitization to pollen molecules is a global health problem. Allergies are thus considered type 1 (IgE-mediated) hypersensitivity reactions to innocuous environmental antigens that is characterized by an imbalanced immune response [16, 17].

### IgE-mediated allergic immune response

In type 1 hypersensitivity reactions, antigen-presenting cells (APCs), mainly dendritic cells (DCs), control the differentiation of naïve T helper cells into effector T cells, such as Th1 or Th2 cells, depending on the nature and source of the antigen [17, 18]. Upon initial allergen encounter, Th2 polarization, a hallmark of allergic sensitization, is triggered by interleukin 4 (IL-4) signalling and characterized by the secretion of Th2-associated cytokines (IL-4, IL-5 and IL-13) [19]. While IL-4 has a key role in the initiation of sensitization, IL-5 and IL-13 are relevant in later stages of the sensitization process as well as the effector phase. IL-5 is mainly involved in airway eosinophilia and hyperresponsiveness, whereas IL-13 mainly contributes to the maintenance of allergic disease by recruiting and activating various effector cells to the site of allergic inflammation [20, 21]. Upon activation by Th2 cytokines, B cells undergo class-switching to produce antigen-specific immunoglobulin E (IgE) antibodies, which prime mast cells and basophils by binding to its high affinity receptor FcεRI. Allergic sensitization is defined by the presence of allergen-specific IgE. Upon re-exposure, allergen-IgE cross-linking causes cell degranulation and release of inflammatory mediators within minutes, leading to the recruitment of other immune cells, and, consequently, triggering the onset of allergic symptoms [22]. Besides its role in allergic diseases, type 2 immunity is also associated with homeostasis and protective immune responses such as wound healing, clearance of parasitic infections and venom resistance [23–35].

### Factors contributing to allergic sensitization

Allergy is generally considered a multifactorial disease, but the individual factors and their respective contribution to sensitization are not yet fully defined. The prevalence of allergic diseases has been associated with a westernized lifestyle—the so-called hygiene hypothesis—but also with environmental and genetic factors. Linkage analysis studies have already revealed allergy-relevant loci, while genome-wide association studies hold the promise for new and reproducible genetic associations with allergic diseases [36]. However, given the multifactorial nature and heterogeneous manifestation of allergic phenotypes, the integration of genetic predisposition data into a coherent picture remains challenging.

The hygiene hypothesis by Strachan, which has been redefined and updated over the years with the continuous emergence of new data, presently postulates that several variables associated with a westernized lifestyle, such as diminished exposure to microbes, environmental changes, medication, diet, parasitic infections and others, influence the susceptibility of the immune system to allergic diseases [37–40]. For instance, in contrast to life in urbanized cities and poor nutrition, growing up near farms and fibre-rich nutrition were classed as beneficial factors leading to immune tolerance [41]. While it is well known that environmental stimuli are directly linked to epigenetic modifications, the field has been understudied in regard to allergic diseases [42]. In summary, experimental and epidemiological findings within the last few decades support the view that the allergic sensitization process is not solely dependent on the genetics of the host and environmental factors, but also on intrinsic features of the allergenic source itself. In addition, epigenetic mechanisms might contribute to the initiation of sensitization and maintenance of allergic diseases [43].

## Initiation of sensitization by allergenic pollen

### The activation of the innate immune system

Besides the known adaptive immunity components involved in allergic sensitization, a superordinate role can be allocated to the epithelium itself since it represents the primary contact site of the human body encountering the pollen. Whether sensitization to pollen-derived allergens occurs at the oral mucosa, the olfactory or corneal epithelium is still a matter of debate; the skin has also been proposed as a sensitization route for allergenic pollen [44–46]. Upon encounter, the pollen hydrates and releases a hydrophilic cocktail consisting of allergenic and non-allergenic proteins and various other bioactive molecules, including lipid mediators, inducing an inflammatory milieu favouring Th2 polarization [47–51].

Epithelial cells and APCs, are endowed with a series of specialized pattern recognition receptors (PRRs), such as Toll-like receptors (TLRs) and protease activated receptors (PARs), which are required to provide first defence mechanisms in order to keep pathogens under surveillance. The vast majority of allergenic tree, grass and weed pollen, including white birch *(Betula verrucosa)*, rye grass *(Lolium perenne)* and short ragweed *(Ambrosia artemisiifolia)*, contain proteolytic enzymes able to disrupt epithelial cells [52–55]. Most identified proteins thereof belong to the family of cysteine, serine and aspartic proteases, and are responsible for the disruption of tight junctions enabling the transportation of allergens across the epithelium. The occurring damage of epithelial cells was observed to be irreversible, but can be blocked by protease-specific inhibitors [56]. However, the question emerges as to what extent these proteases originate from pollen itself (intrinsic origin) or are derived from pollen-inhabiting microorganisms (extrinsic origin) [52]. Purified pollen allergens that are per se non-proteases, such as Ole e 1 and Bet v 1, were also observed to interact with the epithelial barrier [46, 57, 58]. Interestingly, interaction of Japanese cedar *(Cryptomeria japonica)* Cry j 1 and epithelial cells led to the activation of PAR2 and stimulation with Japanese hop *(Humulus japonicus)* increased the PAR2 expression levels on human airway epithelial cells [59, 60]. Direct effects observed for pollen-derived proteases on PAR activation are, however, still lacking but seem to be dependent on the protease class and its abundance within the respective pollen source [55].

Upon stimulation with pollen extracts, epithelial cells release a number of pro-inflammatory cytokines, including IL-1, IL-6, IL-8 and TNFα [55]. Additionally, the secretion of pro-allergic alarmins (*e.g.* thymic stromal lymphopoietin, (TSLP); IL-33; IL-25) favours a Th2-biased immune response and promotes allergic sensitization.

The levels of TSLP are increased in the nasal secretion of patients suffering from allergic rhinitis, but there is no evidence directly correlating TSLP expression and pollen allergen-specific IgE levels, suggesting a more general role of TSLP in the initiation and maintenance of a type 2 inflammatory response [61–63]. In fact, TSLP has been considered a key cytokine driving Th2 polarization through (i) the activation of DCs and macrophages to express OX40 ligand (OX40L), which in turn binds OX40 on naïve CD4^+^ T cells, and (ii) direct induction of IL4^+^ and IL-13^+^ CD4^+^ T cells [64–66]. The pollen-induced secretion of TSLP and the associated type 2 inflammation were observed to be dependent on TLR4 and myeloid differentiation primary response 88 (MyD88), and probably linked to oxidative stress [60, 66–68]. In this respect, stimulation of epithelial cells with pollen extracts from short ragweed, birch, timothy grass and mountain cedar caused elevation in the levels of reactive oxygen species (ROS) [60, 69–71]. Thus, the contribution of oxidative stress to the allergic sensitization is likely mediated by ROS, which up-regulates the expression of PAR2 in epithelial cells, as well as the secretion of TSLP and IL-8 [60, 70]. The latter is a neutrophil chemotactic factor responsible for the recruitment of neutrophils in allergic airway inflammation. Although pollen were observed to induce both TLR4-dependent and independent ROS production, Hosoki et al*.* showed that short ragweed pollen only induced neutrophil recruitment in a TLR4-dependent manner, which in turn facilitated allergic sensitization [67, 69, 71].

As described for TSLP, a TLR4-/MyD88-dependency has also been observed for pollen-induced IL-33-mediated Th2 responses [67, 72]. IL-33 binds and up-regulates its receptor, ST2, expressed on DCs and CD4^+^ T cells, thus triggering Th2 polarization and the expression of the associated cytokines IL-5 and IL-13 [73–76]. TLSP receptor and ST2 double-knockout mice showed a complete ablation of the Th2 response, with decreased Th2-related eosinophilia and specific IgE production, when compared to wild type animals [76]. These observations support the notion that both TLSP and IL-33 cytokines are key players in allergic sensitization to pollen. On the other hand, aluminium hydroxide per se, which is frequently used as adjuvant in most in vivo models of pollen sensitization, facilitates the release of IL-33, making it difficult to assess the individual contribution of IL-33 to allergic sensitization [77].

Another cytokine implicated in the allergic sensitization is IL-25, which has the potential to initiate and activate type 2 innate lymphoid cells (ILC2) and Th2 cells [78]. Increased concentrations of IL-25 were observed in nasal secretion and supernatants from patients allergic to Japanese cedar pollen [79]. However, in a direct comparison with IL-33, IL-25 is less efficient in mediating pollen-induced airway hyperreactivity [80, 81].

In general, the involvement of TLR4 signalling and its ligands, such as the endotoxin lipopolysaccharide (LPS), in the initiation of allergic sensitization is still controversially debated. Although low doses of LPS were shown to be associated with the induction of Th2 cells [82, 83], it is unclear whether TLR4 exerts a more general role in the maintenance of inflammatory responses or is essential in initiating Th2 polarization. Whereas TLR4-deficiency abrogated the ability of birch pollen to activate DCs, stimulation of TLR4-deficient DCs with pollen extracts from Japanese cedar, Japanese cypress, short ragweed or Kentucky bluegrass *(Poa pratensis)* caused up-regulation of maturation markers and induced cytokine secretion similarly to those observed in wild type cells. In vivo*,* only endotoxin-contaminated mugwort pollen *(Artemisia vulgaris)* was able to induce allergic sensitization [84, 85]. In line with these observations, it has been shown that co-administration of TLR4 and TLR2 agonists resulted in a general suppression of the allergic response due to shifting of the immune balance toward Th1 [86, 87]. Taken together, these findings suggest that for some, but not for all pollen sources, activation of DCs, a necessary signal for T cell polarization, occurs in a TLR4-independent manner. In contrast, Dittrich et al*.* demonstrated that innate immune signals are dispensable for the initiation of an inflammatory allergic response to allergens since IL-4, the key cytokine driving Th2 polarization, can bypass TLR4- and MyD88-depedent signalling pathways [88]. Despite the controversies, up-regulation of extracellular TLR2 and TLR4 as well as intracellular TLR3 were detected in the nasal mucosa of allergic rhinitis patients upon allergen challenge [89]. As yet, studies investigating other types of TLRs in the context of allergic sensitization are scarce.

### Origin and essentiality of IL-4

In allergic sensitization, DCs are activated either directly by the allergenic source or indirectly via epithelial cell-secreted alarmins. In turn, activated DCs instruct Th2 polarization by providing three important signals to naïve T cells: (i) antigen-derived peptides presented via MHC-II, (ii) expression of co-stimulatory molecules and (iii) the secretion of pro-inflammatory cytokines and chemokines [90]. Activated DCs associated with Th2 priming show an activation of the transcription factors interferon regulatory factor 4 (IRF4) and GATA-3 [91, 92]. DCs also up-regulate the expression of specific Th2-associated surface markers, including OX40L and the notch ligands jagged-1 and -2 [91–95]. Furthermore, activated DCs secrete CCL17, CCL22 and CXCL13 chemokines and express CXCR5 and CCR7 chemokine receptors, which enable them to migrate to the lymph nodes where they prime naïve T cells to become antigen-specific Th2 cells [93, 96–99].

Apart from the extraordinary role of DCs linking innate to adaptive immunity, the source for the initial IL-4, which is required for efficient Th2 priming, remains elusive. Although basophils, mast cells and NKT cells were shown to produce IL-4, their role remains controversial, particularly in the context of pollen sensitization [100–103]. Once generated, Th2 cells themselves represent the most important source of IL-4. This raises the question of whether IL-4 is strictly required for the initiation of Th2 polarization. IL-33 and IL-25, for instance, are able to mount a STAT6/GATA-3/IL-4-independent Th2 response via the activation of ILC2s and the accompanied secretion of IL-13 [78, 104–107]. Similarly, TSLP induced Th2 polarization in the absence of IL-4 via the involvement of NF-κB and STAT5 [106]. However, the role of STAT5, IRF4, and NKT cells in the context of pollen sensitization is unclear and requires in-depth investigations.

**Fig. 1:**
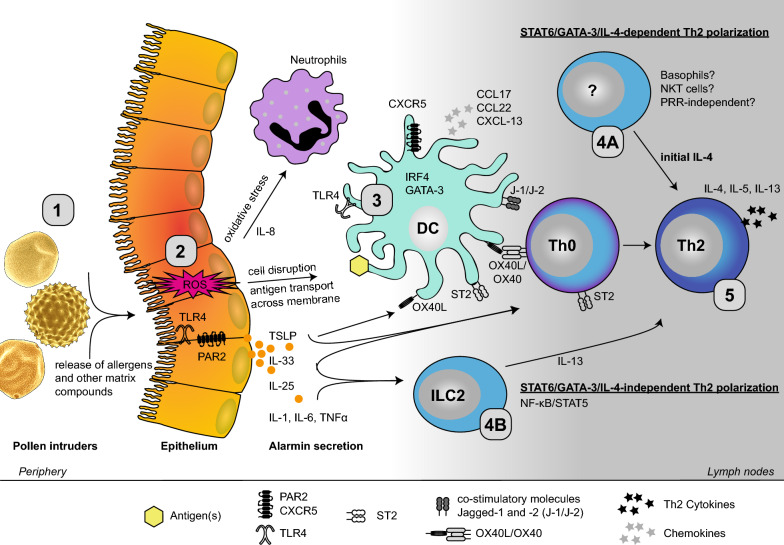
Pollen-induced activation of the innate immune system and Th2 polarization. The initiation of allergic sensitization is a complex network of diverse immune cells, such as DCs, ILC2s and neutrophils. Upon encounter with the epithelium the pollen hydrates and releases its content including allergens and various other bioactive molecules (1). At the epithelium (2), this immunogenic cocktail causes the disruption of the epithelial membrane, activates PRRs such as TLR4 and PAR2, triggers the release of alarmins (TSLP, IL-25 and IL-33), and induces oxidative stress and secretion of IL-8 and other pro-inflammatory cytokines (IL-1, IL-6 and TNFα). In turn, DCs are activated (upregulation of surface markers including OX40L and notch ligands), migrate to the lymph nodes (expression of CXCR5), where they present processed antigens via MHC-II to naïve T cells (3). Th2 polarization occurs either STAT6/GATA-3/IL-4-dependent (4A) or -independent via the NF-κB/STAT5 pathway and the contribution of ILC2s (4B). The origin of initial IL-4 for Th2 polarization is still a matter of discussion; proposed candidate are basophils and NKT cells. Once a Th2 immune response has been initiated, a class-switch of B cells to antigen-specific IgE-producing plasma cells occurs resulting in the sensitization of susceptible individuals to pollen allergens (5). *CCL17, CCL22* chemokine (C–C motif) ligand 17 and 22, *CCR7* C–C chemokine receptor type 7, *CD80, CD86 and CD40* cluster of differentiation 80, 86 and 40, *CXCL-13* C-X-C motif chemokine 13, *CXCR5* C-X-C chemokine receptor type 5, *DCs* dendritic cells, *GATA-3* GATA binding protein 3, *IL-* Interleukin, *ILC2* type 2 innate lymphoid cells, *IRF4* interferon regulatory factor 4, *NF-κB* nuclear factor 'kappa-light-chain-enhancer' of activated B cells, *NKT* natural killer T, *OX40L* OX40 ligand, *PARs* protease activated receptors, *PRRs* pattern recognition receptors, *ROS* reactive oxygen species, *ST2* IL-33 receptor, *STAT5, STAT6* signal transducer and activator of transcription 5 and 6, *Th* T helper cells, *TLR4* toll-like receptor 4, *TNFα* Tumor necrosis factor alpha, *TSLP* thymic stromal lymphopoietin

In summary, the mechanisms involved in the induction of allergic sensitization by pollen are not fully understood. In fact, it seems that different allergenic pollen sources interact with distinct innate receptors and signalling pathways. The involvement of various PRRs, secreted alarmins, and innate immune cells, and the dependency of DCs in driving pollen-induced Th2 inflammatory processes suggest that allergic sensitization to pollen most likely results from particular combinations of pollen-specific signals rather than from a common determinant of allergenicity. An overview of the initiation process of allergic sensitization is presented in Fig. 1 and Table 1.

**Table 1:** Reported immunological events contributing to the induction of allergic sensitization by specific pollen sources and individual major pollen allergens

	Plant family	Plant species		Epithelium + alarmin secretion						Oxidative stress	Neutrophils	TLR4 signaling-		DCs expression of surface molecules			Induction of Th2 polarization	
				Proteolytic activity	Non-proteolytic interaction	PAR2 interaction	TSLP	IL-33	IL-25	ROS induction	IL-8 expression	Dependent?	Independent?	OX40L	ST2	Notch Ligands	Capability	Insufficiency of major allergens
Tree	Betulaceae	White birch *(Betula verrucosa)*		[52, 56, 140, 143]			[61]			[69]		[85]				[95]	[94]	[94]
	Betulaceae	Hazelnut *(Corylus avellana)*																
	Cupressaceae	Japanese cypress *(Chamaecyparis obtusa)*		[56]									[85]					
	Cupressaceae	Japanese cedar *(Cryptomeria japonica)*		[140]					[79]				[85]					
	Cupressaceae	Meditereanen cypress *(Cupressus sempervirens)*		[140]														
	Cupressaceae	Mountain cedar *(Juniperus ashei)*		[144]						[71]								
	Oleaceae	Olive tree *(Olea europaea)*																
	Salicaceae	Black poplar *(Populus nigra)*		[144]						[71]								
Grass	Poaceae	Timothy grass *(Phleum pratense)*								[71]								[115]
	Poaceae	Rye grass *(Lolium perenne)*		[53]						[71]								
	Poaceae	Kentucky bluegrass *(Poa pratensis)*		[53, 56, 143]									[85]					
	Poaceae	Bermuda grass *(Cynodon dactylon)*		[53]						[71]								
	Poaceae	Orchard grass *(Dactylis glomerata)*		[144]														
Weed	Asteraceae	Short ragweed *(Ambrosia artemisiifolia)*		[54, 55, 140, 145]			[66]	[74, 76]		[70]	[67]	[66, 67]	[85]	[66]	[76]		[66, 76]	[116]
	Asteraceae	Mugwort *(Artemisia vulgaris)*					[64]					[84]						
	Asteraceae	Giant ragweed *(Ambrosia trifida)*		[143]														
	Amaranthaceae	Redroot pigweed *(Amaranthus retroflexus)*								[71]								
	Amaranthaceae	Burning bush *(Kochia scoparia)*								[71]								
	Amaranthaceae	Russian thistle *(Salsola kali)*				[60]				[71]								
	Cannabaceae	Japanese hop *(Humulus japonicus)*								[60]								
Allergen	Betulaceae	White birch *(Betula verrucosa)*	Bet v 1		[57]													[94]
	Oleaceae	Olive tree *(Olea europaea)*	Ole e 1		[58]													
	Cupressaceae	Japanese cedar *(Cryptomeria japonica)*	Cry j 1			[59]												
	Cupressaceae	Cypress *(Cupressus arizonica)*	Cup a 1				[75]								[75]			
	Poaceae	Timothy grass *(Phleum pratense)*	Phl p 5							[115]							[115]	[115]
	Asteraceae	Short ragweed *(Ambrosia artemisiifolia)*	Amb a 1														[117]	[116]
	Asteraceae	Short ragweed *(Ambrosia artemisiifolia)*	Amb a 11	[112]														

## The concept of the pollen matrix in allergic sensitization

The question *“why only some environmental proteins cause aberrant Th2-mediated allergic sensitization and others not”* has attracted much attention of researchers. Pollen allergens share specific physicochemical properties, such as hydrophilicity, posttranslational modifications and structural stability that favour the bioavailability and facilitate the antigen uptake by APCs [108–110]. Only a few pollen allergens, including Cyn d 1, Cup a 1 and Amb a 11, exhibit an intrinsic adjuvant activity [75, 111, 112]. Glycan structures on Cyn d 1 from Bermuda grass pollen, for instance, mimic a molecular pattern that binds C-type lectin receptors, PRRs recognizing complex glycan structures [111]. The cysteine protease Amb a 11 from ragweed pollen was shown to initiate a type 2 inflammation via protease-mediated disruption of airway epithelia [112]. Despite the aforementioned characteristics, no generally applicable concept has been put forward to explain the molecular basis of allergenicity, i.e. the capacity of certain molecules to induce type 2 inflammation and specific IgE antibodies [113]. Despite the lack of common characteristics, allergens are still viewed as the main drivers of the allergic immune response since they trigger the typical reactions of the effector phase of sensitization. Nevertheless, growing evidence supports the essential role of pollen-derived components co-delivered with the allergens in the initiation of allergic sensitization [114].

### The immunomodulatory potential of the pollen matrix

Several studies suggested that the allergenic potential of pollen proteins depends on the context of their respective sources. In vitro, purified recombinant Bet v 1 (rBet v 1) could not induce DC maturation compared to a complete aqueous birch pollen extract (BPE). In vivo, BPE, but not rBet v 1, was able to induce Th2 polarization and no differences were observed upon depletion of natural Bet v 1 from BPE [94]. These findings strongly support the view that pure Bet v 1 is a poor immunogen regarding the activation of DCs and the polarization of Th2 cells. We also showed recently that compared to the complete Timothy grass pollen extract, recombinant Phl p 5 (rPhl p 5) was unable to induce IL4-producing Th cells in a short adjuvant-free Th2 polarization in vivo model [115]. Contrasting results have been reported for Amb a 1, the major ragweed pollen allergen. One study showed that the complete ragweed pollen extract, but not purified natural Amb a 1, was able to induce a Th2-biased immune response in mice [116]. On the other hand, Wolf et al*.* reported that purified natural Amb a 1 isoforms were able to induce high IgE titres in mice even in the absence of Alum as adjuvant [117]. It should be mentioned that different immunization routes (i.e. intranasal instillation and subcutaneous injections) were employed in these Amb a 1 studies. In summary, several purified allergens were shown to lack inherent sensitizing potential. However, the respective pollen contexts (*e.g*. aqueous pollen extracts) empower them with their full allergenic potential. According to this hypothesis, sensitization to a given allergen might result from a pre-primed Th2 micromilieu initiated by pollen-derived non-allergenic adjuvant components, a phenomenon described as collateral Th2-priming [118, 119]. Yet, the reason for the high sensitization rates to major allergens such as Bet v 1 remains unclear. It is possible that the amounts and stability of proteins are important requirements for a pollen protein to become a major allergen. In this respect, Bet v 1 is the most abundant protein (10–30% of total proteins) in BPE [94] and its thermal- and proteolytic- stability shown to be modulated by intrinsic pollen compounds [120]. In keeping with this view, allergens would then serve as secondary Th2 targets, i.e. target for IgE antibodies, thus determining the antigenic specificity of the allergic inflammatory response.

### Composition of pollen matrix: intrinsic and extrinsic compounds

**Fig. 2:**
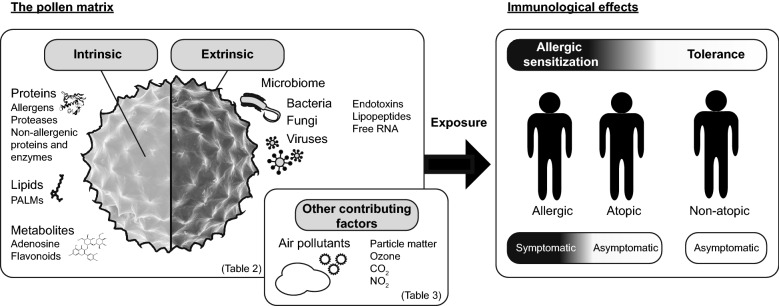
The composition of the pollen matrix influencing the sensitizing potential of allergenic pollen sources. The intrinsic part consists of compounds inherent to the pollen and the extrinsic fraction, includes a prominent microbiome retaining diverse bacterial strains, viruses and fungi. Major immune stimulators can be proteins including allergens with intrinsic adjuvant activities, non-allergenic proteases, but also lipids and metabolites such as PALMS, adenosine and flavonoids. Additionally, climate and exposure to air pollutants shape the composition of the pollen matrix. Details on the immunomodulatory activities of pollen matrix-derived components as well as on the modulation of the matrix composition can be found in Tables 2 and 3. PALMs, pollen-associated lipid mediators; CO_2_, carbon dioxide; NO_2_, nitrogen oxides

Pollen allergens are embedded in a complex and heterogeneous matrix composed of a myriad of bioactive molecules that are co-delivered during the allergic sensitization. The pollen matrix can be divided into two compartments, an intrinsic part consisting of compounds inherent to the pollen, such as proteins, metabolites, lipids, carbohydrates, and an extrinsic fraction, including viruses, aerosols and particles from air pollutants and a pollen-linked microbiome [52, 114, 121–126]. Together these compounds provide a specific context for the allergen, designated as the pollen matrix and proposed as a determinant of allergenicity and Th2 sensitization (Fig. 2).

### Intrinsic compartment

Pollen grains are rich in lipids displaying immunomodulatory effects and contributing to the pathogenesis of pollen allergies [121]. Cypress pollen-derived phospholipids were shown to be presented by MHC-related molecules on DCs to T cells via CD1, an interaction causing T cell proliferation and secretion of IFN-γ and IL-4 in cypress-sensitized individuals, but not in healthy controls [122, 127, 128]. Contribution of invariant natural killer T (iNKT) cells in mediating the effects of pollen lipids was also described for olive and birch pollen in humans and in murine in vitro models, respectively [50, 127].

Several studies described the immunostimulatory activity of non-protein low-molecular weight compounds prepared from pollen extracts. These fractions enriched in various metabolites were able to activate innate immunity signalling and preferentially induced a Th2-biased response (Table 2) [49, 121, 129, 130].

**Table 2:** Immunomodulatory activities of various pollen matrix-derived components promoting allergic sensitization

	Plant family	Plant species	Epithelium		DCs					Neutrophil and eosinophil activation/attraction	Increased reactivity in SPT	Enhanced IgE production by B cells	Airway inflammation
			Barrier permeability	IL-8 secretion	Maturation	CD1d upregulation for interaction with NKT cells	Inhibition of LPS-induced IL-12 secretion	Notch ligands regulation	Inhibition of endosomal cathepsin proteases				
Tree	Betulaceae	White birch *(Betula verrucosa*					Adenosine [129]; PPE1 [51, 135, 136]	LMW fraction [95]	PPE1 [120]	PALMs [132–134]	PALMs [49]		
	Cupressaceae	Japanese cypress *(Chamaecyparis obtusa)*				Lipids [128]							
	Oleaceae	Olive tree *(Olea europaea)*				Lipids [127]							
Grass	Poaceae	Timothy grass *(Phleum pratense)*	Adenosine [138]	Flavonoid isorham-netin [138]	Gram-positive bacteria [126]	Phospholipids [50]				PALMs [132–134]			
	Poaceae	Orchard grass *(Dactylis glomerata)*									Plant virus [123]		
Weed	Asteraceae	Short ragweed *(Ambrosia artemisiifolia)*								Adenosine [116]		PPE1 [137]	Adenosine [116]

*LMW* low molecular weight, *LPS* lipopolysaccharide, *PALMs* pollen-associated lipid mediators, *PPE1* phytoprostane E1, *SPT* skin prick test

Pollen-associated lipid mediators (PALMs), classified into leukotriene-like molecules and phytoprostanes, are eicosanoid-like molecules involved in plant stress responses. PALMs have been identified in aqueous extracts of various pollen sources were shown to attract innate immune cells and to inhibit Th1-differentiation by DCs [131]. Timothy grass- and birch pollen-derived PALMS were shown to attract and activate human neutrophils and eosinophils [132–134]. Upon LPS-stimulation of human DCs, pollen-derived phytoprostane E1 (PPE1) inhibited IL-12p70 production via blocking of NF‐κB and activation of PPAR‐γ, hence suppressing the Th1 response [51, 135, 136]. Further, the protein-free low-molecular weight fraction of ragweed pollen was reported to enhance IgE production by Th2-primed B cells, an effect probably attributable to PPE1 [137]. Gilles et al*.* demonstrated that the low-molecular weight fraction of aqueous pollen extracts induced the expression of Th2-associated notch ligands on DCs [95]. PPE1 was also shown to bind with high affinity to Bet v 1 and to inhibit endolysosomal cathepsin proteases, thus interfering with the antigen-processing machinery in APCs and modulating antigen presentation to T cells [120].

Adenosine has been identified in pollen as a metabolite and immunomodulator with dual properties. Adenosine from BPE was shown to inhibit the production of IL-12p70 by DCs via cAMP signalling. In allogenic co-cultures, BPE-treated DCs from non-atopic donors induced the priming of regulatory T cells. This effect, mediated by pollen-derived adenosine, was less efficient when the DCs in the co-cultures were derived from atopic donors [129]. In vivo, intranasal instillation of adenosine-depleted ragweed pollen extract led to a rapid secretion of Th2-associated cytokines. However, adenosine-depleted pollen extract failed to induce the full allergic lung phenotype when administered to mice that had already been sensitized to ragweed pollen beforehand, showing divergent effects of adenosine: protective during sensitization and pro-inflammatory during challenge. Adenosine-depleted ragweed pollen extract also lost the ability to induce neutrophil and eosinophil migration towards supernatants of bronchial epithelial cells in vitro. Alone, pollen-derived adenosine lacked this ability, suggesting a general effect as cofactor [116]. Functional alteration of bronchial epithelial barriers (ionic permeability and cytokine secretion) was attributed to Timothy grass pollen-derived adenosine and to the flavonoid isorhamnetin. The latter also instructed activated epithelial cells to secrete IL-8, similarly to the complete pollen extract [138]. However, the significance of adenosine in allergic sensitization has been questioned by Mueller et al*.* who quantified adenosine levels in various pollen extracts and concluded that the measured amounts too low to exert a physiological effect [139]. The discrepancy of these results might be explained by environmental factors influencing the adenosine content in pollen.

Pollen contain non-allergenic proteases, either intrinsic to the pollen or extrinsic derived from its microbiome. Proteases in allergenic sources have also been associated with the pathogenesis of inflammatory allergic diseases [52, 114, 140–142]. Pollen-derived proteases can degrade tight junctions and disrupt the airway epithelial barrier, thus facilitate antigen uptake by APCs [143, 144]. The critical role of pollen proteases has been documented in a murine model of asthma in which addition of exogenous proteases to inhaled ovalbumin was necessary to initiate type 2 allergic lung inflammation, whereas the antigen administered alone had no such effect [145].

### Extrinsic compartment

Pollutants in ambient air, such as irritant gases and diesel exhaust particles, ozone, carbon dioxide and nitrogen oxides, do not only affect humans but also plants and their pollen in various ways (Table 3) [125, 146]. Pollutants can influence the composition of the pollen microbiota, induce chemical modifications in allergens, act as adjuvant, damage the epithelial barrier, activate immune cells, and in this way trigger inflammation and promote Th2 polarization [124, 147, 148].

**Table 3:** Modulation of the pollen matrix composition and allergenicity by air pollutants

	Plant family	Plant species	Increase in allergen expression level	Increased PALM release	Increased reactivity in SPT
Tree	Betulaceae	White birch *(Betula verrucosa)*	Ozone [151], air pollutants and bacteria [48]	Traffic-related air pollution [153], air pollutants & bacteria [48]	Ozone [151]
	Platanaceae	Oriental plane tree *(Platanus orientalis)*	Traffic-related air pollution [148]		
Grass	Poaceae	Timothy grass *(Phleum pratense)*	Traffic-related air pollution [153]	Traffic-related air pollution [153]	
	Poaceae	Rye grass *(Lolium perenne)*	Ozone [149]		
Weed	Asteraceae	Short ragweed *(Ambrosia artemisiifolia)*	Ozone [150]		
	Asteraceae	Mugwort *(Artemisia vulgaris)*		Traffic-related air pollution [153]	

*PALMs* pollen-associated lipid mediators, *SPT* skin prick test

In this regard, a correlation between exposure to atmospheric pollutants and the content of allergens as well as of immunostimulatory compounds in pollen was reported [149–151]. Pollen collected from plants growing near roads with heavy traffic and exposed to intense air pollution contained higher levels of PALMs, suggesting a stronger immunostimulatory activity than pollen collected in rural meadow areas [152, 153]. Another study demonstrated a positive association between ambient ozone levels and Bet v 1 sensitization of susceptible individuals [151]. In contrast, the content of PALMs with structural and functional homology to mammalian prostaglandin E_2_ (PGE_2_) was negatively correlated with ozone concentrations. These observations suggest that areas with low ozone levels and high concentration of PGE_2_-like PALMs might facilitate sensitization by promoting Th2 responses, whereas areas with high ozone levels and high allergen concentration might be more prone to induce allergic symptoms in already sensitized individuals. A positive correlation was recently observed between air pollutants and the microbial diversity of birch and Timothy grass pollen, which was further associated with the content of allergen and PALMs.

Although the bacterial species involved in the immunomodulatory effects discussed above have not been identified yet [48], the effect of the microbiota inhabiting pollen deserves further investigation. Its composition is variable and specific for each pollen species [48, 154]. Besides intrinsic pollen-derived lipids, microbial lipids constitute a source of immunomodulators and enhancers of the sensitization process [121]. LPS deriving from gram-negative bacteria has been frequently discussed in the context of allergic sensitization [82, 84, 155, 156]. The presence of endotoxin derived from *Pseudomonas* and *Pantoea* in mugwort pollen extracts was suggested to be a critical factor for the development of airway allergic inflammation in vivo. In contrast, other studies demonstrated that although LPS acts as a strong adjuvant, it does not account for the full sensitizing activity of birch and ragweed pollen extracts [94, 157].

Gram-positive bacteria could also contribute to the extrinsic adjuvant activity of pollen. Supernatants of homogenized *Bacillus cereus* and *B. subtilis* found in high amounts in Timothy grass pollen was able to induce maturation of DCs derived from grass pollen-allergic donors. A co-culture of autologous CD4^+^ T cells with DCs pulsed with grass pollen extract plus supernatants of homogenized bacteria enhanced T cell proliferation, as well as secretion of type-2 and -17 cytokines, compared to DCs pulsed with grass pollen alone, thus, contributing to Th2- and Th17-mediated inflammation [126].

The influence of plant viral infection on the sensitizing potential of pollen remains largely unknown. A small pilot study compared the size of skin wheals after skin-prick tests with Cocksfoot streak potyvirus (CSV)-infected and non-infected Cocksfoot grass pollen extracts [123]. The observed differences suggested that allergic individuals might be more prone to react to virus-infected pollen, which could have implications in allergy diagnosis and treatment.

## The concept of allergen-specific immunotherapy (AIT) in the treatment of pollen allergies

AIT is the only available curative approach of allergic diseases addressing the underlying molecular and cellular mechanisms of the disease. It hereby relies on a constant exposure to allergenic extracts (*e.g.* pollen extracts) making patients tolerant toward the allergen responsible for the occurrence of symptoms [158]. This induction of immune tolerance is facilitated by the immunosuppressive function (direct interaction or release of anti-inflammatory cytokines) of regulatory T and B cells as well as by tolerogenic DCs [158, 159]. However, the latter two have hardly been investigated in context of pollen AIT yet. In the treatment of pollen allergy, AIT is currently performed with pollen extracts that are applied either subcutaneously (SCIT) or sublingually (SLIT). Both treatment options result in an efficient relief of symptoms, although head-to-head SCIT versus SLIT comparative studies are still lacking. At present, the aforementioned treatment choice mostly depends on the patient´s personal preference [160].

There is a clear difference between the United States and European countries regarding the standardization of allergen extracts as well as the usage of adjuvants (*e.g.* aluminium hydroxide, alum adsorption). In contrast to the US, where adjuvant-adsorbed pollen extracts are rarely used, the majority of European countries implemented SCIT products adsorbed to alum in their daily routine [161]. Adjuvants are required to facilitate an adaptive immune response. An additional beneficial effect by alum adsorption of antigens is the “depot effect”, resulting in a slow localized release of the antigens at the sites of administration and, thus, preventing systemic side effects such as anaphylaxis [162]. However, as reported herein, pollen extracts possess their own immunomodulatory efficacy making an adjuvant-free application equally possible. Although the evaluation of recombinant wild-type allergens in clinical trials is scarce, it is apparent they are less effective compared to complete pollen extracts implying the presence of adjuvant signals induce tolerance-favouring mechanisms [163].

A major drawback of current AIT products is the standardization methods of pollen extracts among pharmaceutical companies and batch-to-batch variability. Most extract-based AIT vaccines are standardized toward the concentration of disease-dominating allergen(s), while missing out on the complex nature of extracts [161]. Facing these struggles, it is almost impossible to draw accurate conclusions about the immunoreactivity of pollen extracts regarding the induction of immunotolerance as also reflected by the large variance of responders and non-responders to the treatment, and poor treatment compliance [164]. Other treatment-associated disadvantages are, the limitation in sufficient biomarkers predicting treatment efficacy, a long treatment duration and treatment-induced side effects such as de novo sensitizations to other/minor allergens [165, 166]. In order to overcome these conceptual obstacles it is of utmost importance to point out the necessity of improving current AIT procedures and to provide a sufficient AIT alternative not only able to expedite a more efficient induction of immunotolerance but also a perpetual one.

In light of the present review, and since TLSP, IL-33, and to some extent also IL-25 seem to play important roles in the maintenance of Th2 responses, the concomitantly targeting of alarmins or respective signalling pathways may represent an attractive alternative/addition to current AIT protocols [74, 76, 81]. In addition, the characterization of tolerance-inducing pollen compounds could provide further therapeutic tools to ameliorate AIT efficacy.

## Conclusion

Pollen sensitization results from complex interactions between pollen-derived adjuvants co-delivered with allergens and the innate immune network. These pollen-derived adjuvants are thought to contribute to the generation of a pro-inflammatory microenvironment at exposure sites that primes DCs to favour Th2 polarization in the draining lymph nodes. Due to a multitude of immunological effects reported for various pollen sources upon interaction with the host cells, it seems reasonable to suggest that initiation of sensitization by various pollen occurs via distinct molecular mechanisms, probably also involving pollen species-specific immune adjuvants. The identity of these pollen-derived factors triggering the initial signals for Th2 polarization remains largely unknown. Thus, their identification and evaluation of their role in the initiation of allergic sensitization should to be addressed in future studies. Such findings will provide mechanistic insights into Th2 polarization in allergic sensitization, and pave the way for novel preventive and therapeutic strategies for an efficient management of pollen allergies.

## Data Availability

Not applicable.

## Abbreviations

AIT: Allergen-specific immunotherapy
Alum: Aluminium hydroxide
APCs: Antigen-presenting cells
BPE: Birch pollen extract
cAMP: Cyclic adenosine monophosphate
CCL17, CCL22: Chemokine (C–C motif) ligand 17 and 22
CCR7: C–C chemokine receptor type 7
CD80: CD86 and CD40, cluster of differentiation 80, 86 and 40
CSV: Cocksfoot streak potyvirus
CXCL-13: C-X-C motif chemokine 13
CXCR5: C-X-C chemokine receptor type 5
DCs: Dendritic cells
Fas: CD95, APO-1
GATA-3: GATA binding protein 3
Ig: Immunoglobulin
IL-: Interleukin
ILC2: Type 2 innate lymphoid cells
iNKT: Invariant natural killer T
IRF4: Interferon regulatory factor 4
LPS: Lipopolysaccharide
MHC-II: Major histocompatibility complex class II
MyD88: Myeloid differentiation primary response 88
NF-κB: Nuclear factor 'kappa-light-chain-enhancer' of activated B cells
OX40L: OX40 ligand
PALMs: Pollen-associated lipid mediators
PARs: Protease activated receptors
PPE1: Phytoprostane E1
PR-10: Pathogenesis-related proteins class 10
PRRs: Pattern recognition receptors
PPAR‐γ: Peroxisome proliferator-activated receptor gamma
ROS: Reactive oxygen species
ST2: IL-33 receptor
STAT5, STAT6: Signal transducer and activator of transcription 5 and 6
Th: T helper
TLR: Toll-like receptor
TNFα: Tumor necrosis factor alpha
TSLP: Thymic stromal lymphopoietin
